# The Hospital. Nursing Section

**Published:** 1906-02-17

**Authors:** 


### The Hospital.

### Nursing Section.

Contributions for "THE HOSPITAL," should be addressed to the EDITOR, "THE HOSPITAL" NURSING SECTION, 28 & 29 Southampton Street, Strand, London, W.C.

No. 1,012.—VOL. XXXIX. SATURDAY, FEBRUARY 17, 1906.

### Notes on News from the Nursing World.

### THE QUEEN AND THE JUBILEE INSTITUTE.

AT a meeting held last week the question of a scheme for raising a sufficient sum of money to place the funds of the Queen Victoria Institute on a more satisfactory basis was discussed by Queen Alexandra's Committee, which has recently been formed at the instance of the Queen. Each member is pledged to contribute or raise £100 in the current year to wipe off the deficit, and at the meeting a letter was read from the Queen's Secretary to Lady Cadogan, who acts as President, intimating that Her Majesty desired to contribute £100, and wished him to convey her thanks to the ladies who have consented to join the Committee.

### THE LATE COUNTESS HOWE.

WE greatly regret to learn of the death of Countess Howe, whose name is generally held in honour by nurses who were employed at the Imperial Yeomanry Hospitals during the war in South Africa. While the campaign was in progress our columns testified, from time to time, the immense value of these hospitals, which were splendidly equipped and admirably managed. Lady Howe was the originator of the movement, acted as Chairman of the Imperial Yeomanry Hospitals Committee, was largely responsible for the work of organisation, and was mainly instrumental in obtaining the handsome sum of £170,000 which was subscribed to the Fund. Her energy was unbounded, her enthusiasm irresistible, her tact unfailing, and however much engaged she was, she always found time to give information to nurses and others who sought it. There is no doubt, however, that the attack of neuritis from which she suffered at the close of 1902, following the completion of her labours, was mainly due to the strain imposed upon her for a protracted period. She never, in fact, really recovered from the effect, and she earned, by her patriotism and devotion, a more signal distinction than the Order of St. John of Jerusalem, if it could have been bestowed upon her.

### NEW RULES FOR ADDENBROOKE'S HOSPITAL.

THE new rules and regulations for the training of probationers at Addenbrooke's Hospital, Cambridge, have now come into operation. More details are required from referees than in the past respecting candidates. Personal interviews are insisted upon, no candidate on any account being accepted without one. The matron is of opinion that this is absolutely necessary, and that the suitability of a candidate for nursing can be judged better by seeing her than by any amount of correspondence. The courses of lectures are changed, and the off-duty hours have been readjusted, the former with the view of facilitating the ward work, and the latter with the object of ensuring the nurses the maximum of rest promised in the time-table.

### NURSING ON OCEAN-GOING VESSELS.

WE are now able to give some further particulars as to the conditions on which trained nurses are employed on vessels plying, under the auspices of the Booth Steamship Company, between Liverpool and Brazil. The salary paid is £5 per month, and half-pay during the time the steamer is in port. The voyage lasts about two months, and, as a rule, the vessels are in port for only a couple of weeks. There is no fixed time or length of engagement, but if both the Company and the nurse find things satisfactory, the latter rejoins the vessel when it goes out again. The nurse takes her meals in the saloon with the passengers; she wears her own uniform during the time she is on board or when she goes on shore from the ship. The cases, we understand, are very varied, but sometimes they are those of passengers who have stayed too long abroad and are suffering from complications, the result of frequent attacks of malarial fever. We are glad to learn thus authoritatively that, so far as the Booth Steamship Company are concerned, the trained nurse has become an institution, under conditions which seem to us to be all that could be desired.

### AN ADVENTURESS AS NURSE.

IN the course of the trial, last week, at the Central Criminal Court, of Margaret True Prebble, who also described herself as "Lady Muriel Paget" and "Princess Soltykoff," it was mentioned by the detective inspector, who gave evidence, that the prisoner was "a very clever nurse, and could earn a good livelihood." Inquiries have confirmed the accuracy of the statement that between the periods when she obtained goods under false pretences, making use of well-known names in society, Mrs. Prebble—who avers that she is now only 28—has been connected with several nursing institutions. We are able to say, as a matter of fact, that the adventuress, whose career reads like a penny novelette, was sent by the Leeds Nursing Institution to train at Guy's Hospital on August 14, 1895, under the name of Nina Stolterfoht. She remained less than a month, the then matron having reported her for inferior work and light conduct. In January 1896, as it was stated in evidence, she was appointed, under the same name, second assistant nurse at the Eastern Fever Hospital, Homerton, and was dismissed two months later by the Metropolitan Asylums Board. In 1901 she was attached to the Ipswich Nurses' Home, and is regarded at that institution as "an unpleasant incident." She also subsequently obtained a post as nurse at a sanatorium in Hove, and at the trial the matron of the Winwick Asylum, Lancashire, testified that she was quite recently a member of the nursing staff for a short period, letters coming addressed to her as the "Hon. Muriel Wyndham Paget," in which name she was engaged. She was sentenced to 18 months' imprisonment with hard labour, but as this is the second time she has been convicted of fraud, it would be rash to assume that she will not endeavour, when she is at liberty, to continue her nursing experiences. The moral of the case is that matrons cannot be too careful in the investigations they make respecting the antecedents of the nurses they engage.

### THE ADMISSION OF FRIENDS AS PROBATIONERS

We inserted last week a letter from a nurse who raised the question whether the existence of friendship between two girls should serve as a barrier to prevent them from being accepted as probationers in the same hospital. She does not see the objection from the matron's point of view. When a probationer enters a hospital she soon has opportunities of making friends, and, of course, there is no desire to prevent this. But if two intimate friends were admitted to an institution at the same time it would tend, as we think quite unnecessarily, to increase the cliquism which, under existing circumstances, is often one of the difficulties of the situation; and the most kind-hearted matron is bound to do her utmost to preserve discipline with the least possible friction.

### WATCHING THE DISTRICT NURSE.

To the interest which the Marchioness of Londonderry takes in the Newtownwards District Nursing Society is largely due its prosperous condition, and the fact that it started the year with a balance of £93 in hand. At the annual meeting, which has just been held, Lady Londonderry, who is President and Organiser of the Society, moved the adoption of the report in a speech of considerable length, in the course of which she maintained that the presence of the district nurse in the home diffuses a knowledge of hygiene, and said that those who watched her ministrations learnt how to help in the ordinary care of a sick patient in case of emergency. That is all to the good so long as the learners are content to act only as instructed by the nurse. It is necessary to guard against the danger of encouraging the idea which so often prevails, not only among the poor but in other quarters, that by carefully watching the work of a nurse, another person may qualify herself to act on her own account. The practice of nursing is no more to be acquired by watching a nurse than the practice of medicine is to be learnt by watching a doctor.

### NURSING IN THE SICK WARDS OF CAMBERWELL WORKHOUSE.

In consequence of some information which was sent us last week, being headed "Camberwell Infirmary" it was supposed to refer to that institution instead of to the sick-wards of Constance Road Workhouse. We gladly accept the disclaimer of the Medical Superintendent of Camberwell Infirmary, who says that "Our Infirmary is managed on different lines, and our nurses' leave will favourably compare with that of any infirmary or hospital in the county." But the correspondent to whom we allude certainly shows in a letter which she sends this week that the sick-wards in the Constance Road Workhouse, for which the Camberwell Guardians are responsible, are not sufficiently staffed during the night. While the Guardians deserve credit for the equipment and regulations of the new Infirmary, they ought without delay to bring the other institution in which the sick are received up to date, both for the sake of the patients and of the nurses.

### THE STRESS OF COMPETITION.

The case of Miss Cuddy, a member of the British Nurses' Association, who committed suicide last week by strangling herself with a satin waistband fastened to the bedstead, is very sad and very remarkable. The evidence at the inquest, which included that of Miss Hobbs, Secretary of the British Nurses' Association, that Miss Cuddy was a sober woman and an excellent nurse, showed that the deceased was in great monetary straits, yet had repeatedly refused offers of work at a guinea a week. As Miss Hobbs testified, she declared that "she gave her best work, and would not take less than two guineas a week for anybody." There is no doubt that the feeling that her services were not adequately appraised weighed on the mind of the poor woman and helped to unhinge it; otherwise, in view of the keen competition, we think it is a pity she did not see that half a loaf was better than no bread, and put her very natural pride into her pocket.

### GUARDIANS AND NURSES' SOCIAL GATHERINGS.

The Master of the Oldham Union Workhouse informs us that the matron of the Poor-law Infirmary had nothing whatever to do with the presentation to a medical officer which we mentioned in our issue of January 27. The statement is rather belated, and it does not in the least affect the question raised, which was whether the Guardians acted wisely in claiming the right to concern themselves with issues that are outside the work and the discipline of the nursing staff. The more they, or workhouse masters, refrain from interfering with matters that do not concern them, the more likely they will be to obtain good nurses.

### THE IMPERIAL MILITARY NURSING SERVICE.

We are officially informed that Miss M. E. Brewer and Miss M. Darvill have been appointed staff nurses in Queen Alexandra's Imperial Military Nursing Service. Miss C. K. E. Steel, sister, has been transferred to Wynberg, Cape Colony, from Harrismith. Miss A. B. Cameron, staff nurse, has been posted home to England from Wynberg, Cape Colony, on expiration of tour abroad. Miss J. Hoadley, R.R.C., sister, has been posted to Cambridge Hospital, Aldershot; Miss F. M. Hodgins, sister, to Connaught Hospital, Aldershot; Miss S. I. Snowdon, sister, to Military Hospital, Dover, on their return from South Africa. Miss M. G. Fisher, staff nurse, has been appointed to the Royal Victoria Hospital, Netley. The appointments of Miss A. Ayre, Miss M. Brown, Miss M. Davis, Miss H. B. Derby, Miss C. D. E. F. Dunn, Miss M. C. Johnston, Miss E. K. Kaberry, Miss M. L. Kaberry, Miss C. G. Lees, Miss M. L. Macartney, Miss A. C. Mowat, and Miss A. A. Steer as staff nurses have been confirmed.

### A LEAGUE FOR NURSES OF BIRMINGHAM GENERAL HOSPITAL.

We are informed by Miss M. E. Jones, matron of the General Hospital, Birmingham, that it is proposed to form a League of the nurses who have been trained in that institution. The objects in view are to strengthen the bond of union between the past and present members of the nursing staff by meetings, and the publication of a journal to endeavour to promote the usefulness, honour, and interests of the nursing profession, and to keep a register of members. The annual subscription will be 3s. 6d., which includes a copy of the journal, to be issued at least once a year. Miss Jones, who is herself acting as secretary of the League, will be glad to hear from nurses who trained at the Birmingham General Hospital as soon as possible, and not later than March 31.

### SPECIAL QUALIFICATIONS FOR AUSTRALIAN MATRONS.

The State Education Department at Melbourne has issued its syllabus for cookery, domestic economy, etc., which will form part of the special qualification for hospital matrons. A full course will consist of four terms—each term ten lessons—at a cost of one guinea per term. Nurses may take up this subject during their hospital training or preparatory to entering any institution. Some hospitals, notably the Alfred at Sydney, already make great efforts to interest their probationers in invalid cookery. The prize-giving this year at that institution was quite an event, and the twelve competing trays did great credit to all concerned.

### DISTRICT NURSING AT BRADFORD.

It is now five years since the Bradford District Nursing Association was started, and the report, which was adopted at the annual meeting last month, is of a fairly satisfactory character. The number of patients nursed was 1,116, which included 147 on the books at the beginning of the year; and 42,520 visits were paid by the staff. Of the patients 54 were recommended by the Medical Officer of the Bradford Royal Infirmary, and the nurses paid 12,977 visits to the home patients of the Royal Bradford Infirmary, thus, doubtless, materially diminishing the strain on the resources of that institution. The room for improvement lies in the direction of increased financial support The receipts last year amounted to £1,045, and the cash in hand is just over £70. But to meet the demand for the services of the nurses further subscriptions are needed. The earnestness with which the Mayor and other public men pleaded the cause of the Association at the annual meeting ought to be sufficient to secure an ample augmentation of funds.

### LEEDS TRAINED NURSES' INSTITUTION.

At the thirtieth annual meeting last week of the Leeds Trained Nurses' Institution, it was stated that the staff now numbers 85, and that there are 16 probationers in training schools. During the year 717 cases were undertaken, and 152 were refused from inability to supply nurses. According to the report there are seven nurses now receiving the pension bonus of £10 per annum from the Leeds Trained Nurses' Trust Fund, of the Royal National Pension Fund, and £300 of the profits of the year have been placed to this account.

### WEST BROMWICH UNION INFIRMARY.

The following probationers have received their certificates after three years' training at West Bromwich Workhouse Infirmary:—E. Jones, A. B. C. Turner, S. Shirte, L. Webber, C. Darby, S. Barnbrook, and M. Spooner. The examiner, Dr. Simon, physician to the General Hospital, Birmingham, in his report, dated February 11, states that each nurse was expected to answer a paper containing questions of medical and surgical nursing and massage. Each nurse was also examined *viva voce* on the same subjects. He adds: "I was astonished at and delighted with their proficiency and accurate knowledge. They reached a very high standard indeed in their methods of examination, and I consider that the examination reflects the greatest possible credit on their teachers."

### SOUTH INFIRMARY, CORK.

At the recent final examination held at South Infirmary, Cork, Miss P. Kennedy obtained first place and gold medal in both medical and surgical nursing, scoring the excellent average of 88 per cent. Miss K. O'Brien secured second and silver medal with 84 per cent. Miss M. McCarthy was third with 70 per cent. The examination extended over two days and was very keenly contested. It speaks well for the teaching of the institution that such high marks should be gained by the first three.

### SHORT ITEMS.

On Wednesday last week Miss Emily E. Hoskyn, who has been on the staff at Hebden Bridge Nurses' Home, was married to Dr. J. Newsom Laird, of Macclesfield.—An unusual gift has been made to the Cancer Hospital at Brompton for the benefit of the nurses. In order that they may always know the time—possibly to prevent them from pleading the excuse of not knowing it—a thoughtful clockmaker has put up a large clock in the central hall of the institution. It is, at any rate, a useful present, and will be generally appreciated.

### The Nursing Outlook.

"From magnanimity, all fears above;
From nobler recompense, above applause,
Which owes to man's short outlook all its charm."

### THE DEFEAT OF WRONG METHODS.

It has been a matter of surprise to many who are interested in hospitals and nursing matters, that the people who put themselves most prominently forward, in regard to nurse registration especially, do in fact make so little progress, and have so little weight with the nurses as a whole. The difficulty of the independent observer is increased, too, by the heat introduced into the discussions by the more active of these self-constituted leaders. General regret is expressed that personalities of this kind should be so unnecessarily and so persistently prevalent in the utterances and writings of certain persons, who claim to be influential in nursing affairs. There is evidence of ability, and even of method, on the part of those referred to, but their exhibitions of temper and ill-feeling are so pronounced, as to be discredited in this country at any rate. We have been asked repeatedly for an explanation of these tactics. The explanation probably is, the feeling which often animates small minorities in public affairs, that noise and bluster must be the ruling features of their campaign, for otherwise they would fail to attract public notice. It would further appear that in nursing affairs to-day these so-called leaders regard the nurses in this country as a flock of sheep, and themselves as the shepherd's dogs, who can harry and drive nurses how and where they will. Whatever the explanation may be it is satisfactory to know, that the myth, that the party in question is representative and growing in influence and importance, is now exploded.

Much of the confusion in nursing politics in the past has been due to the circumstance, that every woman, who is entitled to claim that she is a working nurse, is so fully occupied, that she has little or no time to interest herself actively in such matters. This is well known to the few ambitious women, who have fought so hard and so long to obtain the entire control and direction of nursing affairs in this country. They have been left to continue their campaign, up to the present, without much serious remonstrance, because the great body of nurses, and those who really influence them, have felt, that it mattered very little what such self-constituted leaders thought, did, or said. Their conduct recently, however, has excited considerable indignation, not only amongst nurses but amongst the members of the medical profession. Both are coming to realise that if such incursions are not stopped by the exposure of their true aims and motive, the interests of both nurses and doctors may be materially and permanently injured.

People interested in nursing, and the majority of nurses and doctors, being busy people, would be glad to dispose of nursing politics by a cut-and-dried formula, which they can repeat without the trouble of thinking. That is just what it is not good for them, however, to have. What is good for them, and for the public, in these matters is, that they should be compelled to think out the principles and to define them by reason. The principles on which doctors and nurses can unite are the safety, comfort and welfare of the patient, and the promotion of these ends by their skill, devotion and care, at all times and in all circumstances; the insistence upon character as the highest qualification for all attendants on the sick; the exhibition of consideration for both patients, and their friends; and the recognition by the latter of the just claims which a good nurse has to their gratitude and co-operation. First the patient; then the nurse, because of her long hours and anxieties; then the doctor; and then the patient's friends, should rank for consideration in every household where sickness finds a home. The habitual enforcement of these principles when acted upon, will destroy the myth, that the claim of the nurse ranks before all other claims, even in the sick-room. Neither good nurses nor their best friends will ever sanction any such contention.

One of the self-constituted leaders at the meeting of the Royal British Nurses' Association on the 7th instant contended that, having carried a resolution rescinding the proceedings of the meeting on the 17th ultimo, and re-introducing the draft bill in the form defined, the meeting in accordance with the notice was concluded. This transparent objection was promptly disposed of by the chairman. It was characteristic of this objector, who is not inexperienced in the art of packing a meeting, to denounce the *bona fide* members of the British Nurses' Association who voted against her proposals on the 7th inst., as dependants upon the wishes of their masters and mere automatons. It is further characteristic, seeing that she and her supporters were responsible for most of the disorder of which she complained, to describe the meeting as "of the cock-pit type for which the Association is notorious." No doubt this objector would be justified in describing her own and her supporters' conduct in nursing matters, for years past, as being "of the cock-pit type." That is why, perhaps, such influence as they may have once possessed, is slipping away, so that their proceedings, though they may cause some amusement at times, now fail to influence the great body of trained nurses in this country.

### Abdominal Surgery.

By Harold Burrows, M.B., F.R.C.S., Assistant Surgeon to the Seamen's Hospital, Greenwich, and to the Bolingbroke Hospital, Wandsworth Common.

### HERNIA.

*(Continued from p. 272.)*

### Treatment.

The natural tendency of a hernia is to grow larger. This is easy to understand, for every time that a portion of intestine or omentum enters the hernia it stretches the abdominal opening and distends the sac, until at last the opening is so large and the tissues around the sac are so stretched and flaccid that there is nothing to hinder the abdominal viscera from being protruded into the sac. So that, even if no more serious complication ensues, a neglected hernia is practically certain to increase in size, and thus cause greater and greater disfigurement and annoyance to the patient. There is one exception to this rule, and that is umbilical hernia in infants. In this case, if the initial protrusion be very small, the condition tends to undergo spontaneous cure as the umbilical scar contracts. It must not be inferred from this statement that umbilical hernia in infants may be neglected. The defect should always be regarded as a potential source of trouble, and should be carefully treated accordingly.

The general questions which arise in connection with the treatment of a hernia are as follows: Can the hernia be cured by means of a truss or other apparatus? If not, can it be cured by operation? If cure is impossible or improbable, it has to be considered whether an efficient truss can be worn. As a rule an operation is only performed in those cases in which a cure may be expected or in which some urgent complication arises, such as obstruction or strangulation. But there are yet a few cases where it is reasonable to perform an operation in order to render it possible for the patient to wear a truss, and chief among these are the irreducible hernias, for an efficient truss cannot be worn over an irreducible hernia.

The only varieties of hernia in which there is a reasonable hope of effecting a cure by means of trusses are the umbilical and inguinal hernias of infants. If treatment is earnestly and efficiently undertaken from the commencement in these cases, the defect may be cured by trusses. In other cases a cure may be effected by an operation, the aim of which is to remove the hernial sac and to close the defect in the abdominal wall through which the protrusion has escaped. This procedure is known as the "radical cure of hernia." There is yet a large residue of cases in which a cure either by trusses or by operative measures is out of the question. In these the discomforts and dangers inseparable from the presence of a hernia may be lessened, and to a great measure avoided, by the proper use of suitable trusses.

In general it is important in carrying out treatment not to overlook the factors which may cause an increase of abdominal pressure. Thus constipation and gaseous distension of the intestines should be avoided by medicines and suitable dieting. In the case of babies they should, as far as possible, be prevented from crying, and any cough they have will require attention. Rickety children are especially apt to get distended abdomens and to suffer from slight bronchitis, which leads to constant cough. In these a reformation of the diet is an important element in the treatment of the hernia. Circumcision also may be necessary to prevent undue straining during micturition. These are some of the general considerations arising in connection with the treatment of hernia. The particular considerations applicable to certain varieties will now be considered.

### Umbilical Hernia.

This condition is due to yielding of the umbilical scar before it has become firm. If by means of a suitable appliance the abdominal contents are prevented from stretching the scar, the latter will, in the course of a short time, become strong enough to resist the pressure from within the abdomen. As a matter of fact, if the condition is properly attended to from the commencement a cure is practically certain. An efficient method of treatment is to keep a disc of cardboard or poroplastic felt or other material applied to the umbilical region. The disc should be about one inch or one inch and a half in diameter, and about one-quarter of an inch in thickness, and should be covered with a clean piece of linen. It may be kept in place by a firm binder, or, if this is found to be unsatisfactory, by some strapping. At the end of a month or two months the hernia will probably be cured.

The only really successful method of treating umbilical hernias in elderly people is by operation. But the patients are often aged and feeble, and bad subjects for radical cure. At the same time these hernias are especially liable to become irreducible, inflamed, and obstructed, and close watching is needed to prevent disaster, the more so as the patient may complain very little, or not at all, of the hernia, even when obstruction or strangulation is present. To avoid serious complications it is necessary that proper treatment should be employed from the first. A well-fitting umbilical truss should be constantly worn, if radical cure is not performed, and particular pains must be taken to avoid constipation.

If constipation does occur, and is not readily relieved by an injection, obstruction of the hernia should be suspected, and this suspicion will be heightened by any pain, tenderness, increase in size, and hardness of the hernia. Under such circumstances no time should be lost before the medical attendant has had his attention drawn to the patient's condition, for an immediate operation may be required.

### Inguinal Hernia.

In infants and young children inguinal hernias can sometimes be cured by trusses, and nearly always by operation. In adults cure by trusses is very improbable, if not impossible, and operative measures are not nearly so often successful as in children. To cure an inguinal hernia by trusses it is essential *never* to allow the hernia to come down. So long as intestine is permitted to enter and distend the hernial sac, it is manifest that the latter is not likely to become obliterated. Consequently the child must wear the truss at all times—by night as well as by day. It is best to have two trusses, so that when one requires cleaning the other can be applied. These trusses must be covered with rubber or celluloid, so that they can be kept clean easily, and it is unnecessary to remove them while the infant is being bathed. It is, of course, essential to dry the skin underneath the truss and powder it well after the bath. If for any reason the truss has to be removed, the infant should be laid on his back and a finger should be placed on his abdomen immediately underneath the pad, and firm pressure kept on that spot until the same truss or another one has been reapplied. Constant and conscientious endeavour never to allow the abdominal viscera to enter the hernial sac is the essence of successful truss treatment. It is sometimes recommended to use a so-called truss made from a skein of wool. To my mind these wool trusses are not of the least value for the purpose of curing an infantile inguinal hernia, and for this reason their use will not be discussed.

### Trusses.

Some general remarks on trusses may be made here. In the first place, it is essential in acquiring a truss to obtain it under medical supervision from a reputable instrument maker. All sorts of absurd kinds of apparatus are sold as trusses to ill-informed and credulous individuals. It is hardly too much to say that to be of any service at all a truss must be nearly perfect in construction and design; and in this, as in other matters, perfection is not to be found easily. It is certainly foolish to seek for it in some wayside chemist's shop, or by answering an advertisement.

To cure a hernia a truss must be constantly applied. In patients who are no longer infants, and in whom, therefore, the rupture cannot be cured without operation, the truss need be worn, as a rule, only when the patient is up and about, being taken off at night. In this case the truss should not be removed until the patient is lying down in bed; and in the morning it should be re-applied before he gets out of bed. On no account should a truss be applied or taken off except when the patient is lying down on his back.

Any patient who can afford the expense will find it adds considerably to his comfort to have several kinds of trusses according to the various activities which he will have to perform. Thus he may have a bath truss covered with rubber or celluloid to wear during his ablutions, a strong truss to wear during active exercise, and a weaker one for sedentary employment and during the evenings.

A truss should be worn at night if there is much tendency for the hernia to come down at this time, and patients who under ordinary circumstances do not wear their trusses at night should do so if suffering from cough. This is a point to be remembered in nursing certain medical cases where the patient's truss might easily be forgotten.

If a truss causes pain it is almost certain that it fits badly or is unsuitable for the case; it may be that the hernia is not completely reducible, and the pad of the truss consequently presses upon a piece of adherent and inflamed omentum. A common cause of trouble is an ill-fitting inguinal truss. The pad of such a truss ought not to overlap and press upon the os pubis. If it does so it is sure to fail in the control of the hernia, and at the same time will cause pain and discomfort.

Chafing of the skin by the pad may be avoided by keeping both the pad and the skin clean and dry. If, in spite of this, the skin becomes sore, it is probable that the truss requires some alteration, and a visit to the instrument maker is indicated.

### The Nurses' Clinic.

### THE DISTRICT NURSE AND RHEUMATISM.

Cases of acute rheumatism entail considerable work, and the district nurse's first visit can scarcely take less than three hours, even if she knows how to organise and utilise the services of the neighbours who are usually eager to help whenever there is dangerous illness.

The most usual treatment is to wrap the limbs in cotton-wool and flannel bandages. The cotton-wool should be sewn on to strips of flannel or flannelette six inches wide; these are wrapped round the legs and arms and held in place with ordinary 3½- and 2⅝-inch bandages. The hands and feet should be encased in very large boots and gloves made of flannel and lined with cotton-wool, and having a running string at ankle and wrist. In practical district nursing these gloves and boots will be found more lasting and effective than the most accurate bandaging.

The nurse will usually find that pieces of wool have been placed on shoulder, elbow, or collar bone, and are constantly falling away from the spot where they are specially needed, and these fugitive scraps had better be replaced by a large, well-lined pneumonia jacket. A long flannel shirt must be worn; it should be shaped like a child's pinafore, open all the way up the back, and have running strings at neck and wrist. The patient must lie between blankets and the pillows should be covered with flannel. A large cradle must be contrived to keep the weight of the bedding off the legs.

The patient must be thoroughly washed all over every day, an old pair of dry and well warmed blankets being slipped into the bed, and while the washing is going on the blankets that he has lain between must be aired at the fire. No soda should be used for the face, but for all other parts of the body it is needed in the proportion of a teaspoonful to an ordinary toilet basin. The temperature of the water should be 100° F., and as the ablutions are very lengthy the water must be changed or boiling water added to keep up the heat. The hands and feet become black with dirt, and turpentine may be needed for them. It is important to get the hair clean and to keep it so. If the patient is a woman it is most convenient to part her hair in the middle and do it in two plaits firmly tied at the end. The gloves, etc., should be placed at the fire and dried. One of the friends can re-roll the bandages and then hold the patient's limbs to assist the nurse in replacing all the wrappings. When the leg is

**THE NURSES' CLINIC**—*Continued.*

being bandaged the foot should be raised on a pillow. As the heart is often weak the patient's friends should be warned against all sudden movements. The mouth is usually foul and the tongue thickly coated; it should be cleaned with warm Condy, or glycerine and borax, or slices of lemon. The toilet takes fully two hours, and as it is very exhausting to the patient refreshment should be given when the work is half done.

The room must be kept at a temperature of 65° F. The patient's temperature commonly reaches 103° or 104° F., but delirium is rare; milk and a farinaceous diet is required; meat must be entirely abjured until permission to eat it is given by the doctor; white fish is often allowed as soon as the patient begins to get better.

There is always great fear of pneumonia, and to relieve the lungs the patient should not always lie on his back, but be turned slightly on one side and be supported in that position by pillows. The patient must be absolutely forbidden to get out of bed, and a very flat bed-pan should be used.

The duration of rheumatic fever is variable, as relapses which cannot be traced to any error of treatment often occur. The patient should be strongly advised to continue to wear flannel under-garments, even when all apparent danger is over. I have nursed the same woman with rheumatic fever four times in eight years.

If there is great pain in one special joint it should be bathed with warm water and soda (one teaspoonful to the pint) wrapped round with hot wool, flannel, mackintosh, and more wool. By the next morning the pain will probably be gone.

In cases of chronic rheumatism the patient must be advised not to give way, but to struggle up every day and work, and insist on using the joints. If this is done perseveringly complete stiffness of the joints can generally be held at bay.

Hot vapour baths often alleviate pain and stiffness. In other cases a hot alkaline bath (twelve ounces soda to thirty gallons water) may give relief. The patient should remain in the bath fifteen minutes, supported by a knitted hammock and with a pillow placed against the metal bath. She must then be laid between hot blankets and given hot milk to drink. The baths have to be persevered in for a considerable length of time.

Painful joints may also be rubbed with camphorated liniment, or in some cases opium and belladonna may be ordered.

Six and three-quarter yards of flannel or flannelette will be required for boots, gloves, pneumonia jacket, and outer bandages, and all but the last of these must be lined with cotton-wool. For the wide inner bandages seven yards of each material will be needed.

### Incidents in a Nurse's Life.

Contributions to this column are invited.

### THE SON AND HEIR.

The night bell rang! I rubbed my eyes, for I knew that it was my turn to go. The summons came. I made a hurried toilet, for I had only just passed my examination (C.M.B.) and felt the responsibility and dignity of my new position. In seven minutes I was ready and my pupil waiting for me; we started. We found the house as only district nurses can discover their destination in those miserable dwelling places in the East End, by some sort of instinct.

Our patient was a big German woman, who hardly understood English. She had five girls, and was most anxious for a boy. I decided it would not be long before baby was born. We were busy preparing the bed *à l'anglaise* when the door opened to admit a short thin man, whose eyes, head, and sallow complexion could belong to no one but a Japanese. He was dressed in a long frock-coat, the tails of which flapped against his legs as he rushed in. He wished to remain in the room, explaining that in Japan they had always been accustomed to a German midwife, who had allowed him to be present at the birth, and also that he was a chemist, "as gude as any docteur." All this was accompanied by many gesticulations and flappings of the frock-coat!

"I vill help, I vill help," he cried again. Politely I declined his services, and requested him to leave us. At last he sorrowfully went, but reappeared at short intervals with large cups of weak tea—just an excuse for coming in. Poor little man! I was sorry for him. He loved his frau, and she was in the hands of strangers.

He left us in peace at last—at least so we thought when a quarter of an hour had passed without another cup and saucer being handed in. Suddenly, however, he burst in once more, his hands extended, his sallow face full of trouble.

"Oh, you vill not vash the babby in cold watter, vill you? De German midwife did and the babby took the chill"—here his voice broke.

I comforted him, but turned him out and barricaded the door with a broken chair. The baby arrived ten minutes later, and was a boy. There was great rejoicing; in fact the credit was all given to the nurse *anglaise*, "who did everything so nice."

Before washing him I allowed the father to put his hands in the water, in order to convince him that his son would not get a chill. He was quite satisfied and loud in his thanks.

The next day, when a pupil paid the usual visit, the father refused to allow her to touch the baby! He washed and dressed it himself; and nurse declared that he was as careful in his handling of the infant as any woman could be. When I went in to say "good-bye" on the tenth day the little man told me his history. He had been in a good position in his own country, but had been persuaded to try England. How he had come over to set up as a chemist. But everything had gone against him. All their earnings had been spent, and he was obliged to take a post in a chemist's shop. I believe nearly everything had been pawned except the frock-coat kept for this special occasion. How often it is that one finds humour and sadness so interwoven.

I often laugh over the remembrance of my German patient and her little Japanese husband, and how the English nurse was considered more efficient than the German midwife, who had never presented him with a boy, and who had washed her babies in cold "watter." But I sigh when I realise the sadness of the lives of those who have "seen better days"—the love that helps them to bear their burdens, and the power of endurance they are forced to sustain.

### Across the Seas: A Missionary Hospital at Sierra Leone.

BY ONE OF THE SISTERS.

The Princess Christian Cottage Hospital in Sierra Leone, West Africa, was built as a result of the earnest united efforts of Bishop and Mrs. Ingham in 1892. Bishop Ingham resigned in 1897, and the then Canon Missioner, Canon Taylor Smith, was consecrated Bishop. Bishop Taylor Smith resigned the episcopate on his appointment as Chaplain-General to His Majesty's forces in 1901, and was succeeded by the present Bishop, E. H. Elwin, D.D., who had been the Secretary of the Church Missionary Society and Principal of Fourah Bay College in Sierra Leone. The hospital is worked by three English sisters, one of whom is generally on furlough in England, an English doctor, and four of the native girls who are being trained as nurses. There is one large ward for women and children—natives—containing twelve beds and three cots; and one other small ward in which the white people are nursed, fitted up with mosquito curtains over doors and windows.

### The Coloured Patients.

When I first went into the large ward, three years ago, I was surprised to find such a beautiful fresh airy place. A nice dark polished floor and green painted walls, which looked so fresh and cool coming in from the glare of the hot sun outside. There are nice iron-framed beds and spring mattresses, from England, and pretty white quilts with a large Maltese cross in turkey-red worked in the centre. For a minute a visitor might almost think herself in one of our English hospitals, but only for a minute, for on looking above the quilts, instead of the white pasty faces of our English sick ones, there are the jet-black faces of the native patients, with their white shining teeth and their black short curly hair. When these coloured people have got over the strangeness of the hospital they make splendid patients. They have unbounded faith in "the white sisters," and will let them do anything for them, and in their funny broken English they will tell their different ailments, generally ending up with "Pain bad, Sister, bad for true," and shake their woolly heads as if their last hour had come. Doctors and Sisters soon grow wise in West Africa, and trust more to the charts and nurses' reports, aided by their own observations, than to the patients' own story.

### Early Work.

I must try and describe one of our busy days in the hospital. At 5.30 a.m. the night nurses call us, bringing a lighted lamp, for it is quite dark. We pull our mosquito curtains aside and feel for our shoes, which we put on immediately, for fear the "jiggers" may get into our bare feet. The "jiggers" are tiny little insects which burrow into the feet, generally by the side of the big toe nail, and there lay their eggs. The native girls are very skilful in extracting these "jiggers"; but many times we get poor little neglected children with toes eaten away by these troublesome creatures. The night nurse leaves her written report to be read, the spelling and grammar being most original. By six o'clock it is broad daylight. There is no dawn slowly creeping on, or twilight in the evening, as in England, but the daylight comes and goes very quickly. These early morning hours are the best part of the day, and we hurry off for our early morning cup of tea and bread and butter before going to the ward. On entering the ward we are greeted on all hands with "Good morning Sister, Carbo, Carbo," and then straight away we begin morning prayers, for ours is a Mission hospital, and while the utmost is done by human skill for the sick bodies of the poor women, at the same time we try to influence them spiritually. After morning prayers there is much to do before the doctor comes at 7 o'clock. Each patient wants to tell you her tale of the night passed; there are beds to make, helpless patients to wash, daily dressings to do, and the ward to be dusted and swept, and all to be made clean and sweet and tidy for the Medical Officer.

### The Crowd Outside.

All this time, since before daybreak, there has been a crowd collecting of the halt, lame, blind, and sick, and when the doctor has finished his morning round in the wards, he goes down to a little consulting-room, near the out-patients' hall, and one by one all the sick people go in to him and tell him their woes. There are many funny scenes enacted in that little consulting-room during the morning, and many sad ones, too. Sisters and nurses are kept busy all the time dressing wounds, giving the various kinds of treatment ordered by the doctor on the patient's paper, and doing all the dispensing of the medicines ordered. We try to get in a word for our religion as we bandage limbs, put plasters on, draw teeth, open abscesses, sew up cuts, and do the hundred and one different things that are needed. The young girls we especially invite to a Bible class which the Matron has started on Thursday evenings, and from which we have had great encouragement. The patients are great believers in magic, and often we find little black charms tied on to the woolly head of some sick one to charm away an ulcerating sore, or to stop toothache or ear-ache.

### A Well-earned Rest.

So the busy morning flies along, and at last we have finished the 100 to 120 patients who have had to be treated. Every minute now it is growing hotter and hotter, and we are glad to hurry through our midday meal and go and lie down on the top of our beds while the sun is at its greatest heat. At three we are up again refreshed after the rest and bath, and are in the ward giving medicines and seeing that the nurse left in charge—our staff nurse—has done everything properly. The patients are as well looked after as any patients could be in our English hospitals. At six o'clock it suddenly becomes dark again. We have evening prayers, and at eight the night nurse comes on duty. Sister goes her last round for the night, leaving due instructions to be called if necessary. We are very proud of our black probationers, though we never dare tell them so; they make excellent nurses, for they are so gentle and quiet; but they need constant watching and overlooking for fear they get slack. A good work is being daily patiently carried on at this little hospital amidst many difficulties—great tropical heat, tropical insect life, and malaria. In the Medical Officer's report for last year he reported that out of 238 in-patients treated 148 were cured; out of 76 chloroform cases 68 were relieved; 9,383 visits were paid by out-patients and 3,956 out-patients' dressings were done.

### "The Hospital" Convalescent Fund.

The Hon. Secretary begs to acknowledge, with grateful thanks, the receipt of £1 from Miss L. J. Attree, and 2s. 6d. from Nurse Ruel.

### The Bishop of London on Babies.

A tea and entertainment was given under the auspices of the St. Clement's Maternity Home to upwards of 500 mothers attended during the past year at Fulham Town Hall on Tuesday last. In addition to this there was a baby show, the prizes going to the best-kept and healthiest babies. The mothers began to assemble before five, each bearing a spotlessly clean and highly decked baby. A most attractive-looking tea was awaiting them, and whilst it was going on the judging of the babies took place. Tea and clearing away took a good while, but by eight o'clock, mothers, children, and a good number of fathers, were ready for the speeches and entertainment. The Bishop of London arrived punctually, and after greeting Miss Heatley, shook hands cordially with all the nurses and said a few kindly words to each.

The Rev. R. Free, Vicar of St. Clement's, Fulham, presided, and in his opening speech congratulated Miss Heatley upon the gathering he saw before him, and bore testimony to the very excellent work done by all connected with the Home.

The Bishop of London, who was greeted with much applause, began his speech: "Ladies, gentlemen, and babies!" then went on to say that he had not known beforehand whether he was to face 500 fathers, or 500 mothers, or 500 babies, or all mixed together as he found them, but anyhow he knew he was going to enjoy himself, because he was coming amongst his neighbours. During the winter he was obliged to live at London House for his work's sake, but he always looked upon Fulham as his home. At this stage the Bishop plaintively remarked that he always noticed his voice woke up the babies and that at mothers' meetings whenever he spoke the babies always talked back. There were certainly good grounds for this complaint, which was received with much laughter. The Bishop proceeded to observe that he had lately been commenting in public about the birth-rate, and it was a very great pleasure to him to see that it seemed more flourishing. But what he wanted to know was were the mothers feeding and bringing up these children properly? ("Let *me* have a chance," said the Bishop pleading to a vociferous youngster in front.) It was all very well for the nurses to see that the children were properly looked after at birth, but were they properly fed afterwards? Of course he was only a poor bachelor, still he knew that it was not a good thing to give whisky or beer to an infant, or beefsteak to a child of six months. Speaking seriously, he thought that there was no more grave question concerning the country's welfare in the future than the proper feeding of the children. He hoped that the mothers would learn all they could from the expert ladies in their midst, and would bring up the future generation in the proper way. He came to them as their Bishop, and he wanted to say a few words from the heart. What were the uses of anxiety? Fathers and mothers both suffered great anxiety at the time when children were being brought into the world. Now, anxiety had three uses. It was very good for us; speaking as one who had a great deal, he said that anything was better than a dead calm. Then anxiety threw us upon God. We then felt the need of God. And it also seemed to fit us for the most excellent joy. A woman forgot all the troubles of child-birth "for joy that a man was born into the world." Many of them had great difficulties in living, but these were preparing them for the joy that no one could take from them.

At the conclusion of the speeches a concert took place, and during the interval, at 9.30, Mr. Hayes-Fisher distributed the prizes, consisting of smart dolls or very desirable toys.

### Appointments.

Bridgwater Hospital.—Miss Edith Murphy has been appointed sister. She was trained at Cardiff Infirmary, where she was afterwards staff nurse. She has since done private nursing at Bristol, returning to her training school to do holiday duty.

Cama Hospital, Bombay.—Miss M. Hilson has been appointed lady superintendent. She was trained at the London Hospital, Whitechapel, and has since been a member of the Army Nursing Service Reserve in South Africa, has done district work among the women and children in cantonments, Potchefstroom, and been lady superintendent of Ramsay Hospital, Naini Tal, India.

Carnarvonshire and Anglesey Infirmary, Bangor.—Miss P. B. Woodhead and Miss Grace B. Douglas have been appointed charge nurses. Miss Woodhead was trained at Huddersfield Infirmary, and Miss Douglas at the Royal Alexandra Infirmary, Paisley.

City Hospital, Fazakerley, Liverpool.—Miss Gwenllian Evans has been appointed deputy-matron. She was trained at the Fever Hospital, Middlesbrough, and Leeds Union Infirmary. She has since held the posts of ward sister and night superintendent at the City Hospital, Netherfield Road, Liverpool.

East London Hospital for Children, Shadwell.—Miss Constance Mundy has been appointed sister. She was trained at the Victoria Hospital for Children, Chelsea, and the Royal Derby Infirmary.

Fordingbridge Cottage Hospital, Hants.—Miss E. Dale has been appointed nurse matron. She was trained at the Royal Infirmary, Edinburgh, has since done private nursing for the Leeds Trained Nurses' Institution, and been charge nurse at the Union Infirmary, Kington, Herefordshire.

Monsall Fever Hospital, Newton Heath.—Miss Elsie Benians and Miss Sarah Beattie have been appointed sisters. Miss Benians was trained at Bethnal Green Infirmary, London, where she has since been staff nurse and sister. Miss Beattie was trained at Monsall Fever Hospital, and the Royal Infirmary, Liverpool. She has since been staff nurse at the Royal Infirmary, Liverpool.

National Hospital, Queen Square, Bloomsbury.—Miss Edith A. Sordy has been appointed sister. She was trained at Torbay Hospital, Torquay, and has since been staff nurse at the National Hospital, Queen Square, Bloomsbury, where she has obtained a certificate for massage, and also done temporary sister's duties.

Royal Ear Hospital, Soho, London.—Miss Gertrude Dorkins has been appointed staff nurse. She was trained at West Ham Infirmary, and has since been attached to Woodford Nursing Institute, and temporarily nurse at the General Hospital, Walthamstow.

Royal National Orthopædic Hospital.—Miss E. Fairbairn has been appointed home sister. She was trained at the London Hospital, and has since been temporary sister at the Royal Chest Hospital, City Road, London, and district nurse at Hastings. She holds the certificate of the Central Midwives Board.

Sheffield Royal Hospital.—Miss A. Rastall has been appointed sister of the theatre and men's surgical ward, and Miss V. M. Durram has been appointed sister of the outpatient department. Miss Rastall was trained at Sheffield Royal Hospital, and has been sister of the out-patient department in the same institution. Miss Durram was trained at Sheffield Royal Hospital, where she has since been staff nurse.

### Everybody's Opinion.

[Correspondence on all subjects is invited, but we cannot in any way be responsible for the opinions expressed by our correspondents. No communication can be entertained if the name and address of the correspondent are not given as a guarantee of good faith, but not necessarily for publication. All correspondents should write on one side of the paper only.]

### OVERWORKED PROBATIONER NURSES.

"A PROVINCIAL NURSE" writes: Referring to the "Outlook" last week and to the account in THE MIRROR *re* sad suicide of a nurse at a Dublin hospital, your correspondent "A. M. M. B." objects to the sweeping rider of the jury that the twelve hours' duty are too long. Certainly it appears too long to those who do not really understand the routine of hospital life; but I wonder what they would have to say of night nurses who are on duty daily fourteen and often sixteen hours—my own experience. In numbers of small hospitals throughout the country, say, of about thirty beds, there is only one sister for day duty and only a nurse and one probationer for night duty. But even more important in the matter of guarding against breakdowns than long hours is that the matrons of our hospitals, both large and small, should look upon it as part of their duty to inquire closely into the general health of their nurses. If they did, I feel sure that many a young nurse would be spared a great deal of nervous indisposition, and certainly we should have fewer cases of health failure. It is strange that nursing authorities should overlook this fact, for it becomes more pronounced each day that nursing is a career only for the survival of the fittest. We nurses do not object to the amount of hard work the profession demands of each who enters the ranks, but when one has to labour under the disadvantages of a nervous headache for months, which is quite enough to depress even the brightest of spirits and unfit them for their work, really one cannot marvel at a mind becoming deranged and a sad end following. Again I say that if a matron would always interest herself in the welfare of her nurses, and encourage them when not feeling quite up to the standard as regards their health to go and tell her all, that she might, if possible, treat, or at least advise them, there would be a decrease in the number of women who enter upon their work with such earnestness, but alas! leave later with all hopes of a useful career vanished. Some may add that if this suggestion were followed that nurses would always be ailing, but I feel certain that a nurse conscientious in her work would never condescend to play so mean a part. I do not consider, of course, that every matron is guilty of this negligence. Quite the reverse. My plea is to those who are apt to look upon nurses as mere machines, from whom to extract as much work as possible, and if any cannot stand the strain do not feel concerned, as "there are plenty more waiting for a chance!" This is what most hurts a girl fresh from home and all its loving surroundings, when she finds that she is looked upon as a "means to an end," whereas when entering into the work she thought that the best points of her character and her womanhood were about to be developed and encouraged.

### THE RESPONSIBILITIES OF A SISTER.

"H. M. E." writes: In last week's issue of THE HOSPITAL I read an article headed "The Responsibilities of a Matron." May I be allowed to say a few words on "The Responsibilities of a Sister"? No doubt the responsibility increases in the upward movement, from pro. to staff nurse, from staff nurse to sister; but the summit of our ambition, also the summit of our responsibility, lies in the word "matron." However, it is of the sister I wish to speak. Take, for example, a ward of 30 beds in a fever hospital, and allow the sister a staff nurse and two junior nurses to assist her. She is entirely responsible for every detail, both in treatment of patients, in ward management, and instruction of nurses. Some of the nurses may not be all that is required, and may not even wish to be so. On the other hand, the sister may get nurses "after her own heart," but no sooner is this accomplished than they are moved to another ward. Each nurse should be held responsible for her share of the work, whether it is ward management or treatment of patients, the sister to be referred to in any doubt or difficulty, and to also assist in carrying out the more important duties until she is satisfied that her staff nurse is capable of fulfilling these duties without her continual supervision. I also maintain that when a nurse is at fault she should take the consequences of her carelessness, provided that the matter in hand is one she thoroughly understands; and again, if it were something she did not understand, she should be reproved for acting without reference to the sister. Nothing is more annoying than to hear a nurse say: "I thought that was right." In our training we are taught "not to think." I know of one or two hospitals where the sisters (who, by the way, must hold a three years' certificate and have had previous experience as a sister) are held responsible for every detail that the nurses may choose to forget; also for the wardmaid who may have failed to eradicate a stain or a speck of dust from the highly-polished floor. The sister "should see" everything done. What reward does the sister reap for all this vigilance? Does she enjoy any of these little privileges so dear to hospital folk, a little more home comfort, or a little more freedom of action than the nurses, whose responsibilities are in this case nil? The answer is, No! Her off-duty time is doled out to her without any choice on her part; her duty and off-duty time is the same as her junior nurse, although she holds a higher position and is a trained, experienced nurse, she must feel she is still a probationer when she is treated as such; neither is it good for discipline. This is the exception, not the rule. No doubt the matron prides herself on being just in the extreme by treating all members of her nursing staff alike, with one exception—herself. I speak from experience. I have been in seven hospitals. Two were carried on under the principles I have described, and the prevalent feeling was discontent. Thus I feel that the responsibilities of a sister could almost bear comparison with the responsibilities of a matron.

### NIGHT NURSES' HOURS.

"A LOVER OF JUSTICE" writes: I was interested in the notes mentioning night nurses' hours. I am now in the fifth month of night duty "solus" at a suburban hospital of 25 beds, and the hours on duty are from 9 P.M. to 9.30 A.M.: needless to say, the idea comes from North Country infirmary training. My previous experience under Bart.'s and Guy's nurses has never exceeded three months' night duty. My opinion is that lengthy night duty, if it is to be done as it should be done, demands uninterrupted self-sacrifice, and plays havoc on the one who will accede to its demands; and, failing this, it demoralises.

"REFORMING DAY SISTER" writes: While "A. M. M. B." makes a good case for hospital authorities, the other side of the question is also not unworthy of consideration. To begin with, to be up at night is unnatural. Therefore to compare the amount of work to be done at night with what is done in the day is obviously unfair. One nurse is, so to speak, running on level ground with Nature's law; the other is running up hill against Nature's law. Then the average day nurse is asleep by 10.30 P.M., and even though she may have to rise at 6 A.M., she has had a good rest. What night nurse can count even three times a week, five hours a day unbroken sleep? Opinions on all subjects differ, but to my mind the possibility of sewing or studying does not compensate for loss of rest. And then, unfortunately, nurses, like other women, are not free from "nerves." I know they ought to be, but the fact remains that they are not. And which of us cannot recall some ghastly shocks among our night duty experiences? It may have been a sudden death; it may have been a delirious patient; it may only have been the cat on the kitchen dresser and a jug of milk on the floor. But how long it took to bring our hearts back to the normal beat! And the following day, when we had at last coaxed sleep to our weary pillow, did not cats and patients and jugs all mix together in one weird dream, and rob our sleep of any quality of rest? I have done with night duty now, and, I hope, for ever; but my sympathies are entirely with night nurses. And with the Dublin jury I emphatically say that 12 hours' night duty is too long.

### TRAVELLING WITH A DIPSOMANIAC.

"I. M. M." writes: I feel I must write what I think about the nurse who relates her experiences in travelling with a dipsomaniac in your columns. Surely to travel with a flask of brandy in her bag and to let her poor patient know that she had it was most cruel and wrong. She does not seem to have provided any food for her patient, and when the poor woman fled from her to the refreshment-room never tried to persuade her to have some coffee or bovril. Most likely if she had got her to take some nourishment she would not have been obliged to call in the aid of a policeman, and so have made her patient furious. The patient's anger was perfectly justified. The nurse appears to have thought of nothing but her own discomfort on the journey, and so scarcely upheld the honour of her high calling as a nurse. If she had looked on the poor woman as a patient to be helped, suffering from a most insiduous form of poisoning, instead of condemning her as a very bad sort of sinner, she might have succeeded better on the journey, and had the satisfaction of feeling that she had helped a sister woman in distress. Happily other nurses have acted very differently. I know one who has had several of these cases, and has been able to restore some of her patients to their friends perfectly recovered. She does not agree with this nurse, but thinks that if the cause is removed that first made a woman take to stimulants, if the poison is thoroughly got out of her, and plenty of good food got in, and she is treated with proper kindness and sympathy, and made to feel that she is among friends, there is no reason why a woman should not recover as thoroughly as a man can from this complaint.

### THE TRAINING AT EDINBURGH ROYAL INFIRMARY.

THE TREASURER AND CLERK of the Royal Infirmary, Edinburgh, writes: A few words of explanation are called for in connection with the paragraph in your issue of February 3, headed "The Training at Edinburgh Infirmary." For the past seventeen years—since October 1888, to be exact—every nurse accepted for ordinary training at this institution has had to pass through a three years' course before receiving a certificate. A certain number of "pupil probationers" have been taken each year—*i.e.* women sent by private nursing and other institutions—these receiving two years' training but no certificate, and it was to that class that the Board of Managers' decision of July 1903 referred. Outstanding agreements had naturally to be fulfilled, and the fact that these had all been completed was recorded in the manager's report for 1905. In justice to the many nurses in all parts of the world who hold the certificate of the Royal Infirmary of Edinburgh, I trust you will find space for this letter in your next issue.

[The paragraph in question had reference to the decision that in future every probationer at the Edinburgh Royal Infirmary should train for three years. We had no idea of conveying the impression that the ordinary training of the general body of probationers was less than three years. We have reason to believe that it is, in fact, excellent.—ED. THE HOSPITAL.]

### OFF-DUTY HOURS AT A LONDON WORKHOUSE.

"A NURSE" writes: The statement you make in reference to off-duty hours at Camberwell Infirmary last week should have been applied to the sick wards in Constance Road Workhouse. It is an undoubted fact that the cases are extremely heavy, and helpless in many ways. There are from 45 to 55 patients in each ward, and three or four nights in a week, from 7 to 10.30, a night nurse has her own patients and those in the ward below to look after—in all 99 cases. As there is a flight of stone stairs between the wards, the single nurse is necessarily bound to run up and down very often, and is fearfully anxious when away from either. In one ward there are about 20 epileptics, many of them almost lunatics, and some times two or three dying, and these, I maintain, should not be left without a nurse. In the morning a single nurse has besides other patients to wash, three who are quite helpless, and I consider that one nurse, worn out with a long night of duty, ought not to be expected to do this without some one to help in the lifting. The strain and anxiety are, in fact, more than one person ought to be required to bear, and I hope that the publicity given to the matter will be the means of bringing about a change for the better.

### SODA-WATER AND BURNS.

"A DISTRICT NURSE" writes: May I be allowed space in your columns to ask the opinion of my fellow workers on the use of strong soda-water being applied to a burn or scald, with a subsequent dressing of cream and olive oil? A short time ago I was called in to a child while on my rounds who had scalded his hand and arm. However, the grandmother arrived on the scene first, and being already in possession, would allow of no interference. She had placed the hand and arm in hot soda-water "to bring out the fire," as she said; then a dressing of cream and olive oil was applied. The wounds healed up in a marvellously short space of time, leaving only the faintest scar. Milk was always used for the removal of the dressing, care being taken not to break the blisters.

### The Nurses' Bookshelf.

INSTRUCTIONS TO MIDWIVES. Compiled by the Midwives Supervising Committee of the Corporation of Manchester. (London: Scientific Press, 28 and 29 Southampton Street, Strand, W.C. 6d. net.)

THIS pamphlet opens with a definition which is certainly not to be found in Johnson's Dictionary. "A midwife is a woman certified under this Act (Midwives Act, 1902). Until she is on the roll and certified a person is not a midwife." Having laid this principle down clearly, the compilers go on to extract from the Act the regulations affecting the midwife's work, and to give details of what is required of her with respect to her own person, her dwelling place, and her tools. The instructions are clear and practical, and such as can be easily taken in by any sensible woman. It is noticeable, however, that the authors lay great stress on the use of perchloride of mercury almost exclusively as a disinfectant. Lysol is not mentioned except as a douche, while mercury is enforced for cleansing the patient externally, for the midwife's appliances and her hands. She is to cleanse her hands by washing and soaking them in a basin of perchloride of mercury solution, 1 in 1,000, for not less than two minutes, and to repeat the proceeding as often as is necessary. Yet no hint is given her of the possibility of mercurial poisoning, which would not be very remote in the case of a busy woman who attended a large number of cases. Also it is seen in practice that when the hands are sterilised with lysol no further lubricant is necessary, thus avoiding the danger of using a pot of glycerine of perchloride, into which the fingers are probably dipped over and over again. The directions as to the care of the mother after labour and of the child are excellent.

SIMPLE ADVICE TO A YOUNG MOTHER AND HOW TO FEED HER BABY. By E. H. YOUNG, sister of a London hospital. (London: Scientific Press, 28 and 29 Southampton Street, Strand, W.C. 6d. net.)

IN this little book useful advice is given in the simplest of terms for mothers of the class who cannot afford the services of experienced persons for their babies. The first part concerns the health of the mother before the birth of the child, warning her what to expect as to constipation, morning sickness, etc., and enumerating also the requisites for the confinement. The second part contains advice on washing, dressing, feeding, and sleep of the baby, and suggests what is best to be done in the case of small ailments. For example: "If the baby has continual diarrhœa alter something in its diet, and clothe it with more flannel." "Do not persist in giving it food whilst there is severe vomiting." There can be no doubt that the distribution of the book among "district" patients, especially if a few comments were made on the contents, might result in more effectual care being given to the babies by ignorant, because untaught, mothers.

DON'TS FOR MOTHERS AND NURSES. (Leeds: Reynolds and Branson, Limited. 6d. the set of six.)

SIX amusing little caricatures on postcards of the methods (or want of method) of bad nurses. One represents a "lady" wheeling a mailcart, in which an unfortunate child is sitting asleep, with his head bent down over the strap of the chair till it touches his knees, thus illustrating the motto, "Don't wheel out your baby in this position." Another card pictures a very stout mother nursing a marasmic-looking baby, by the side of a large round table covered with various favourite articles of food and drink—tinned salmon, beer, potatoes, etc. The accompanying advice is, "Don't give baby a taste of everything you have yourself." They are clever sketches, but unhappily the last to see the point of them would be the very persons who indulge in these practices.

THE ENGLISHWOMAN'S YEAR BOOK AND DIRECTORY, 1906. (London: Adam and Charles Black. Price 2s. 6d. net.)

FOR the twenty-sixth year that extremely useful volume, "The Englishwoman's Year Book and Directory," has made its welcome appearance. Several departments are much enlarged, and all the old ones are maintained and made thoroughly up to date. The "employments and professions" of course include nurses, and we remind our readers who contemplate taking up the work of sanitary inspectors that full details of the nature of the duties, etc., may be found in its pages. It is stated that the demand for women inspectors is still steadily on the increase, and that the work of those already appointed has proved, on the whole, successful. The competition is, however, likely in any case to be keen. Miss Emily James, the editor of this "Year Book," fulfils her task with great judgment and an unusual sense of the necessity for the observance of proportion.

THE DREAMER. By LUCAS CLEEVE. Second impression. (Digby Long and Co., 6s.)

LUCAS CLEEVE gives in John Page the dreamer a consistent character study of a man cursed with the artistic temperament. From the outset of his career it spells failure for himself and trouble to those who are dependent on his exertions. The setting of the story is America. This should attract readers who prefer a less unconventional environment than that provided by the ordinary British novelist.

### Novelties for Nurses.

(BY OUR SHOPPING CORRESPONDENT.)

### NEW WASHING FABRICS.

IN these days, when by far the greatest wear and tear of washing fabrics is sustained at the hands of the modern laundry, it is essential that the fabrics chosen by nurses for their uniforms should be alike durable in colour and material. Mr. Christopher Williamson, of 91 Edgware Road, London, W., seems to have solved this problem by producing some charmingly pretty as well as durable materials for the purpose. Two specialities of the establishment are a grey lustre Gingham at 7¾d. and some Wynburg Regatta at 9¾d. per yard, which look as if they would never wear out. The Wynburg Regatta is made in various shades, a pale grey and a butcher blue suggesting at once a suitable background for the professional white apron. In strong washing Oxfords there is a nice range of colours, and the wide linens in white and colours at 1s. 1d. per yard are splendid value, as are also the Veronica dress Oxfords, in stripes of various colours, at 6¾d. per yard.

### Notes and Queries.

### REGULATIONS.

**The Editor is always willing to answer in this column, without any fee, all reasonable questions, as soon as possible.**

**But the following rules must be carefully observed.**

1. **Every communication must be accompanied by the name and address of the writer.**
2. **The question must always bear upon nursing, directly or indirectly.**

**If an answer is required by letter a fee of half-a-crown must be enclosed with the note containing the inquiry.**

*Dispensing.*

(149) I am a certificated dispenser (Apothecaries' Hall) and am now desirous of getting a post as assistant dispenser in a hospital. I should be willing to give any spare time to work as a probationer. Can you advise me how to find such an appointment?—*B. G.*

You had better advertise for what you require.

*Liverpool.*

(150) I should feel greatly obliged if you can tell me of a union infirmary in or near Liverpool where they would take me as a probationer, and how long would my name be required to remain on their books before I could expect to enter the infirmary. I am twenty-one years of age?—*H. H.*

You are too young to enter as a probationer, twenty-three years being the usual age. You might, however, write for particulars to the matron of the Mill Road Infirmary, Liverpool, where probationers are received at twenty-two. Before a vacancy occurs you may have reached that age. Probationers are received at twenty for two years' training at the Wirral Hospital for Children, Birkenhead.

*Lotions.*

(151) I should be glad if you would answer the following: (1) How much carbolic acid 1 in 20 does one take to make 1 in 40, 1 in 60, and 1 in 80, and how much water does one add. How much perchloride of mercury in 1,500 does one take to make 1,000, 1 in 2,000. How much water does one add? What is the easiest way to calculate quickly the different solutions of lotions?—*Puzzled.*

Any good text-book on nursing or dispensing would give you the information you desire.

*Children's Hospital.*

(152) Will you be kind enough to give me the name of any children's hospital or convalescent home, or any institution of the kind, into which I might enter. I am only twenty-one years of age, and therefore not yet eligible for the usual training. Will you please state whether wages are given or otherwise?—*E. C.*

You might write to the matron, Paddington Green Children's Hospital, salary £12 first year. Also Belgrave Hospital for Children, Clapham Road. No salary first year. East London Hospital for Children, Shadwell, £10 first year.

*Club.*

(153) I am a permanent nurse, and should be very glad if you could tell me of any club I could join that I could go to when off duty, either on week-days or Sundays; W. or W.C. preferred.—*Nurse R.*

Write for particulars to the Hon. Secretary, the Midwives' Institute and Trained Nurses' Club, 12 Buckingham Street, Strand, W.C.; St. Andrew's House Club, Mortimer Street; the 20th Century Club, Stanley Gardens, W.

*Central Midwives Board Examinations.*

(154) Do you know of a book covering the requirements of the Central Midwives Board Examinations? I do not want a midwifery book. Seven years ago I trained at Glasgow and got my certificate to act as midwife and nurse, but neglected to apply for registration before the time expired, and now find I must go in for another examination. I have plenty of midwifery books, but would like to know what is required for the Central Midwives Board.—*Nurse S. Y. S.*

See "How to Become a Certified Midwife," by Dr. Appel, published by the Scientific Press, Limited, 28 and 29 Southampton Street, Strand, or write to the Secretary of the Midwives' Institute, 12 Buckingham Street, Strand, W.C., for advice.

*Canada.*

(155) Can you give me any information as to the prospects of English nurses in Canada, especially in the north-west? I am anxious to join friends in Weyburn, North-West Territory, but would like to feel sure of work before going out.—*Nurse E. T. T.*

It is quite impossible to feel sure of work in Canada unless you have friends there who will promise to keep you going.

Canadian nurses are so well trained that there is little need for English ones.

Can you tell me . . . if there are openings for private nurses either in Canada or in the United States, or do Americans prefer their own trained nurses to those trained in England?—*Nurse C.*

See answer to *Nurse E. T. T.* The same remarks apply to the United States.

*Training.*

(156) Will you kindly tell me if there is any hospital where they would take me, having had some training in a small general hospital. I should like to know if they would give me a salary?—*C. R.*

Probably several. Consult "How to Become a Nurse," published by the Scientific Press, Limited, 28 and 29 Southampton Street, Strand, W.C., and write to several suitable hospitals, giving details.

*Stains.*

(157) Can you tell me anything that would remove stains of nitrate of silver solution, 1 per cent., from cotton sheets. The stains are large, and the articles have been washed?—*M. G.*

The only thing to remove such stains is a deadly poison, the use of which we cannot recommend.

*Homes.*

(158) I should feel much obliged if you would tell me if there is any home or institution where I could place two respectable young women, orphans, without means, one suffering from chronic rheumatism, the other from anæmia in an advanced form. Would a small sum yearly or a donation help?—*E. D.*

You might write to St. Peter's Home and Sisterhood, Mortimer Road, Kilburn, or St. Elizabeth Home, 59 Mortimer Street, W., or St. Marylebone Home, 61 Weymouth Street, Portland Place, W. Small payment required. "Burdett's Hospitals and Charities" gives a full list of such homes and institutions.

*Australia.*

(159) Can you tell me where I could get information as to posts vacant in Melbourne, or Victoria, either as monthly nurse or midwife?—*Nurse.*

You might write for particulars to the lady superintendent of the Melbourne Trained Nurses' Home, 384-386 Albert Street, East Melbourne, and to the lady superintendent of the Melbourne Nurses' Home, 6 Parliament Place, East Melbourne, Victoria. But Australia has plenty of maternity nurses.

*Study.*

(160) Do you know anyone who would answer questions of nurses who are studying alone—by post?—*Nurse M. A.*

You can easily find out by advertising.

*Enteric.*

(161) Will you kindly inform me if it would be possible for me to get about three or six months' experience in the nursing of enteric fever, and if so where? Would it be necessary to go to a fever hospital? I am not desirous of coming in contact with scarlet fever or diphtheria.—*M. R.*

We are afraid it would be impossible to make special arrangements to work in the enteric ward only.

*Mission Work.*

(162) I have just completed three years' general hospital training. I should like to take up some Christian work that would still require the services of a nurse. Could you advise any home or church that would employ me? I am a member of the Baptist Church. I should like to go abroad. Would there be work for me among English people?—*F. M. E.*

You might write to the Secretary of the Baptist Missionary Society 19 Furnival Street, Holborn, E.C., to any of the large missionary societies, or to the Hospital Nurses' Union, 5 Cambridge Gate, Regent's Park, N. W.

*Woman Unable to Work.*

(163) Is there a home where a woman of 34, an orphan and penniless, unable to work through valvular disease of the heart, could gain admittance?—*Somersetshire.*

You might write to St. Mary's Home, Highmore, Parkstone, Dorset, or St. Cyprian's Home, 31 The Grove, Hammersmith. She might be suitable for the British Hospital for Incurables, Streatham, or the Royal Hospital for Incurables, Putney Heath. Admission is by votes.

**Handbooks for Nurses.**

| | Post Free. |
|---|---|
| "How to Become a Nurse: How and Where to Train." | 2s. 4d. |
| "Nursing: its Theory and Practice." (Lewis.) ... | 3s. 6d. |
| "Nurses' Pronouncing Dictionary of Medical Terms." | 2s. 6d. |
| "Complete Handbook of Midwifery." (Watson.) ... | 6s. 4d. |
| "Preparation for Operation in Private Houses." ... | 0s. 6d. |

Of all booksellers or of the Scientific Press, Limited, 28 & 29 Southampton Street, Strand, London, W.C.

### For Reading to the Sick.

### THE HIGHEST LESSON OF ALL.

"Beloved, let us love one another," says St. John,
Eagle of eagles calling from above :
Words of strong nourishment for life to feed upon,
"Beloved, let us love."

Voice of an eagle, yea, Voice of the Dove :
If we may love, winter is past and gone;
Publish we, praise we, for lo! it is enough.

More sunny than sunshine that ever yet shone,
Sweetener of the bitter, smoother of the rough,
Highest lesson of all lessons for all to con,
"Beloved, let us love."

*Christina Rossetti.*

There is no lack of points for devout meditation in the great resurrection parable of the miraculous draught of fishes.

First, consider that it was God's working in Providence which revealed to the devout heart of St. John the presence of God. It was not that the strange mystery which shrouded the Sacred Form was removed, but that St. John saw instantly, in the course of events, the presence of his Lord and his God. Many books have been written to convince doubting hearts of the great truths of a loving, intelligent, personal God. No doubt all these books have their use. But there is a surer proof of God's presence than any argument can bring, a proof which is most convincing, most clear, to those who are, like St. John, full of reverence and full of love : the proof is the daily working of our common lives. Who that meditates on past years, nay, even on one past day, cannot discern again and again the guiding hand of God?

You would be an Apostle—it is well : only the power which can accomplish miracles is love. Not orthodoxy, not sentiment, valuable as each of them are, but love; or put it in other words—not faith, not hope, but charity. And the test of this love shall be constant self-sacrificing, patient work for God. Be it ours, then, daily to pray for an increase of real charity; not only to check uncharitable words, but to strangle all thoughts which are contrary to love : daily pray to the Spirit of Love that He would pour into our hearts "that most excellent gift of charity" that loving, not in word, neither in tongue; but in deed and in truth, we may be in very truth workers together with God.

All excellence shall live; all work which is good is eternal. It may be buried, hidden unknown here; but in so far as it is excellent, it will be eternal. Nor does this only mean that each one will contribute the result of labour or skill to the joy of Heaven. Surely it applies also to moral excellence : to those who have been gentle and tender even as a nurse it will be said, "Bring all this sweetness and affection, and let it shine evermore upon the inhabitants of the happy land."

*T. Birkett Dover.*

Grief will come, and loss will come,
Saddening many a morrow;
But through all, though often dumb,
Blessing even sorrow,
Love, that knits the souls of friends,
Makes for all divine amends.

*A. Matheson.*

